# Assessing the use of a drought-tolerant variety as adaptation strategy for maize production under climate change in the savannas of Nigeria

**DOI:** 10.1038/s41598-021-88277-6

**Published:** 2021-04-26

**Authors:** Abdullahi I. Tofa, Alpha Y. Kamara, Bashir A. Babaji, Folorunso M. Akinseye, Jenneh F. Bebeley

**Affiliations:** 1 International Institute of Tropical Agriculture (IITA), Oyo Rd, P.M.B. 5320, Ibadan, Nigeria; 2 Department of Agronomy, Ahmadu Bello University, P.M.B. 1045, Zaria, Nigeria; 3 Centre for Dryland Agriculture, Bayero University, Kano, Nigeria; 4 International Crop Research Institute for Semi-Arid Tropics (ICRISAT), Kano, Nigeria; 5 Department of Meteorology and Climate Science, Federal University of Technology, PMB704, Akure, Ondo Nigeria

**Keywords:** Climate sciences, Environmental sciences

## Abstract

The Decision Support System for Agricultural Technology Transfer (DSSAT) was used to quantify the impact of climate change on maize yield and the potential benefits of the use of drought-tolerant maize variety over non-drought tolerant variety in savanna ecological zones of Nigeria. Projections of maize yields were estimated for three locations representing different agro-climatic zones and soil conditions, in the mid-century (2040–2069) and end-century (2070–2099) under representative concentration pathways scenarios (RCP 4.5 and 8.5) against the baseline period (1980–2009). Relative to the baseline period, the ensemble Global Circulation Models (GCMs) predicted significant increase in minimum and maximum temperatures and seasonal rainfall across the sites. In the mid-century, ensemble GCMs predicted temperatures increase between 1.7–2.4 °C for RCP4.5 and 2.2–2.9 °C for RCP8.5. By end-century, the temperature increases between 2.2–3.0 °C under RCP4.5 and 3.9–5.0 °C under RCP8.5. Predicted seasonal rainfall increase between 1.2–7% for RCP4.5 and 0.03–10.6% for RCP8.5 in the mid-century. By end of century, rainfall is expected to increase between 2–6.7% for RCP4.5 and 3.3–20.1% for RCP8.5. The DSSAT model predictions indicated a negative impact on maize yield in all the selected sites, but the degree of the impact varies with variety and location. In the mid-century, the results showed that the yield of the non–drought tolerant maize variety, SAMMAZ-16 will decline by 13–19% under RCP4.5 and 19–28% under RCP8.5. The projection by end-century indicates a decline in yield by 18–26% under RCP4.5 and 38–47% under RCP8.5. The yield of the drought-tolerant variety is projected to decline by 9–18% for RCP4.5 and 14–25% for RCP8.5 in the mid-century and 13–23% under RCP4.5 and 32–43% under RCP8.5 by the end-century. The higher temperatures by both emission scenarios (RCP 4.5 and 8.5) were primarily shown to cause more yield losses for non-drought-tolerant variety than that of the drought-tolerant variety. There will be 1–6% less reduction in yield when drought-tolerant variety is used. However, the higher yield reductions in the range of − 13 to − 43% predicted for the drought-tolerant variety by the end of the century across the study areas highlighted the need to modify the maize breeding scheme to combine both tolerances to drought and heat stresses in the agro-ecological zones of northern Nigeria.

## Introduction

Maize (*Zea mays* L.) production in Nigeria has been increasing rapidly both in terms of hectarage and production. This is evident from the annual production of above 10 million tons on area of 4.9 million hectares in 2018^1^. According to Ezeaku et al.^2^, Nigeria required an estimated 50% increase in maize production to meet the increasing demands over the coming decades. Despite increase in current production, Nigeria’s average maize yield of 2 t ha^–1^ is among the lowest of the top 10 maize producers in Africa^1^. The bulk of the maize is produced in the Northern region where the savannas have favourable conditions required for maize growth^3,4^. Major constraints to maize production in the Nigeria savannas are poor soil fertility^5,6^, *Striga hermonthica* parasitism^7,8^, drought and unsuitable temperatures^9^. Frequent drought and high temperatures resulting from climate variability and change negatively impact maize production in the Nigeria savannas.

Generally, crops have an optimal temperature for performance, beyond which yields rapidly decline^10,11^. According to Liu et al.^12^ the optimum maize‐growing temperature in Sub-Saharan Africa (SSA) is 25 °C. Although maize is usually considered as a warm season crop it is actually more sensitive to high temperature stress as compared to other crops^13^. For every degree of an increase in global mean temperature, average maize yields are projected to decrease by 7.4%^14,15^. Maize is particularly vulnerable to heat stress during the reproductive stage^16–18^. Findings from Bita and Gerats^19^, indicated that heat stress during flowering and grain filling stages resulted in decreased grain number and weight, leading to low crop yield and quality. There is already undeniable evidence that Nigeria has witnessed increasing trends in the annual temperatures^20–24^. Climate projections for the coming decades in Nigeria predicted there will be a temperature increase of between 2 and 2.5 °C across the country by 2050^25^, with a rise of up to 3.2 °C under a high-resolution global climate change scenario within the same century^10^. Likewise, the regional temperature variations are also expected, with the highest increase (4.5 °C) projected in the northeast by 2100^11^. Shiru et al.^26^ revealed an increase in rainfall in the south-south, southwest, and parts of the northwest with a decrease in the southeast, northeast, and parts of central Nigeria. They projected annual rainfall change in the range of − 7.5% to + 27% by end-century, thereby, projecting for future drought and flooding in the country. Drought is a major abiotic stress limiting maize production and productivity in SSA, contributing between 44 to 58% grain yield reduction in West and Central Africa^27,28^. Many places in the Guinea savannas of Nigeria now experience yearly drought that often coincides with flowering period of maize crops and consequently leads to poor grain yield or total crop failure^29^. It is being speculated that the frequency and intensity of drought would intensify in the years ahead in response to climate change^30^. Such extreme conditions of high temperature and drought will have negative impact on agriculture and food security in Nigeria which largely dependent on rain-fed food production systems^31^.

Future evidence of climate change impact highlights the need to explore adaptation strategies that can reduce the negative impacts. Some studies suggested improved crop management practices like the deployment of drought-tolerant varieties^32,33^ could improve yields, and reduce loses due to potential climate change effects^34,35^. The use of drought-tolerant varieties have been reported to reduce maize yield loss due to climate change. Cairns et al.^36^ reported that drought-tolerant maize varieties are resilient to the effects of high temperature in SSA. A study by Tesfaye et al.^33^ showed significant yield reductions of local maize varieties by 21, 33 and 50% when temperature increased by 1, 2 and 4 °C, respectively under hotter and drier climate change scenarios in South Africa. In comparison to the local maize varieties, the yield advantage of either drought, heat or combined drought and heat tolerant varieties were increased under both baseline and future climate scenarios.

The use of crop models to assess climate change impact and adaptation measures remain limited in West Africa^35^. The combined use of Global Climate Models (GCMs), crop simulation models and statistical downscaling techniques are the primary tools available to assess climate change impact on maize growth and yield^37,38^. Several studies have shown the impact of climate change on maize using different climate change scenarios and crop simulation models in SSA^38–41^. In Nigeria, some studies have examined impacts of climate change on maize production and productivity resulting in several adaption strategies being promoted to reduce the negative effects of climate change^2,42–45^. Although several drought-tolerant maize varieties have been released for cultivation in the Nigerian savannas, no study has compared the impact of climate change on these varieties relative to the non-drought-tolerant ones. The objective of the study was to assess the impacts of climate change on the yield of drought-tolerant maize variety as compared to a non-drought-tolerant variety in the Nigerian savannas using the CERES-Maize (DSSAT) model.

## Materials and methods

### Description of the study area

**Figure 1:**
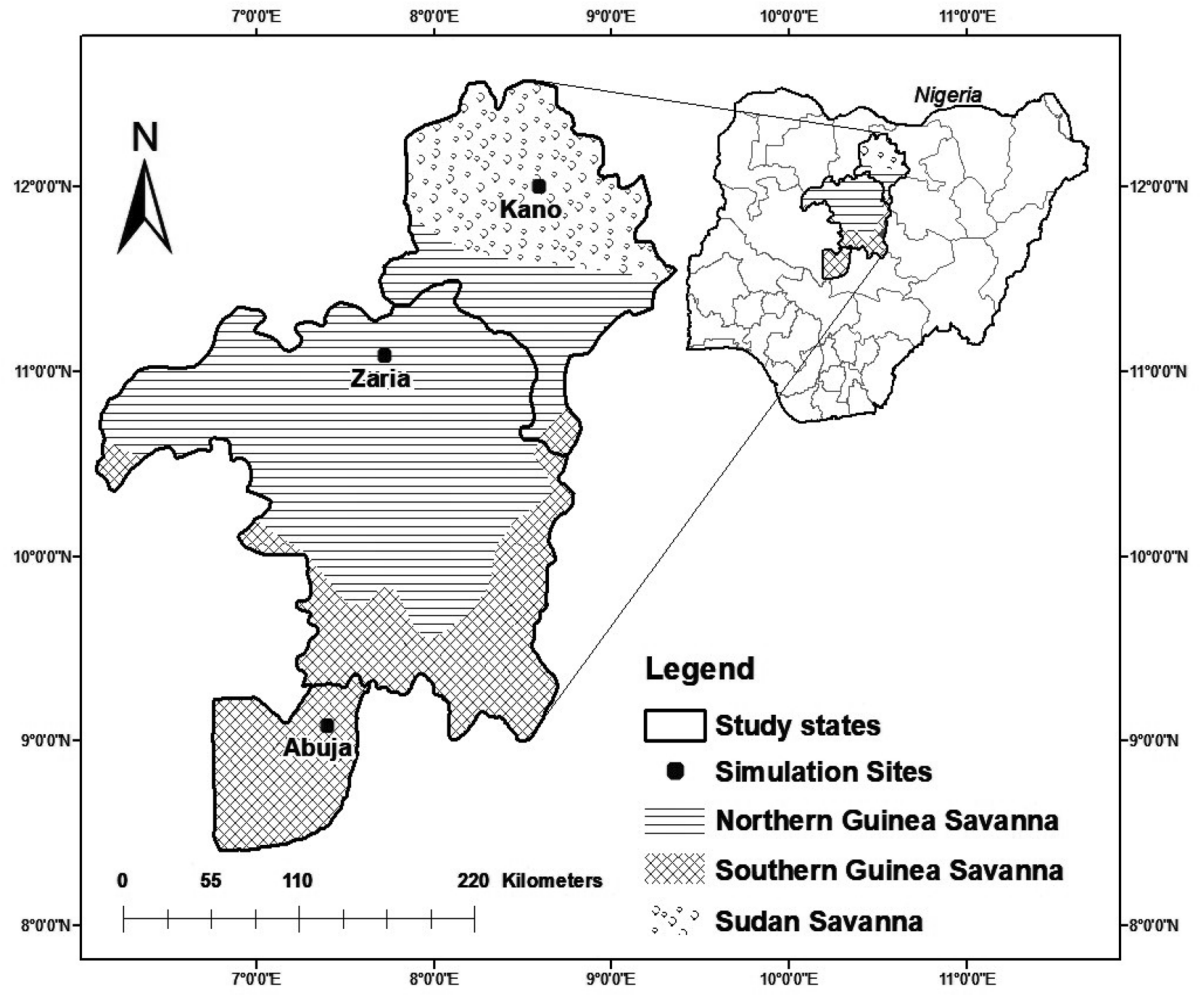
Map of the study area. This map was created using ArcGIS [GIS software] version 10.4.1. Redlands, CA: Environmental Systems Research Institute, Inc., (Copyright ESRI, 2015). ESRI. (2015). Copyright 2015. ArcGIS 10.4.1 Computer software. Redlands, CA: ESRI.

The study was carried out in three representative sites of the southern Guinea, northern Guinea and Sudan savannas of northern Nigeria (Fig. 1), which largely covers the maize cultivation area of Nigeria^3,4^. Kano represents the Sudan savanna (SS) zone and lies 484 m above sea level (a.s.l.). The SS is characterized by single long dry season followed by a rainy season that extends from May to October. The long-term (1980–2009) average seasonal rainfall at Kano was 753 ± 171 mm year^−1^. The average number of rainy days was 57 ± 9, with average maximum and minimum temperatures of 33.7 and 20.0 °C, respectively. Zaria represents the northern Guinea savanna (NGS) maize production zone which is characterized by a mono-modal rainfall pattern^46^. The long-term seasonal rainfall was 998 ± 133 mm with average number of rainy days of 63 ± 9, and the mean maximum and minimum temperatures were 31.6 and 19.2 °C, respectively. Abuja representing the southern Guinea savanna (SGS) production zone had unimodal rainfall pattern with longer rainy days (average of 108 ± 18) which extends from April- October. The long-term average seasonal rainfall is 1541 ± 270 mm year^−1^ with an average maximum and minimum temperatures of 32.4 and 21.1 °C, respectively.

### Soil properties of the study area

Soils in Kano in the SS of Nigeria are highly weathered and fragile with low clay content^47^. The dominant soil class of the site is Alfisol according to the USDA soil taxonomy^48^. In Zaria in the NGS, the soils are classified as leached ferruginous tropical soils with a high clay content and overlying drift materials^49^. This soil type is classified as Typic Haplustalf according to USDA soil taxonomy^50^. The dominant soils in Abuja in the SGS are generally shallow and sandy in nature^51^ and categorized as Ferric Luvisols^52^. The characteristics of the soils used for model calibration was earlier reported by Tofa et al.^53^.

### Baseline and future climate scenarios

The baseline climate parameters including daily rainfall, maximum and minimum temperatures, and solar radiation for 31 years (1980–2010) were obtained from the Nigerian Meteorological agency (NIMET) archives for the three studied sites. The protocols developed by the global Agricultural Model Intercomparison and Improvement Project (AgMIP) team^54^ was used to generate the future climate scenarios (RCP4.5 and RCP8.5) for mid-century (2040–2069) and end-century (2070–2099) using the delta-based method. The future climate scenarios under RCP4.5 and RCP8.5, assume elevated CO_2_ concentration of 499 and 571 ppm, respectively, compared with the current 380 ppm. The future daily rainfall, minimum and maximum temperatures were generated by perturbing the daily baseline data using delta factor method^55,56^. For the study, we used four contrasting, bias corrected GCMs from Fifth Coupled Model Inter-comparison Project (CMIP5) including CESM1-CAM5 (National Centre for Atmospheric Research, USA), CSIRO-MK3.6.0 (Commonwealth Scientific and Industrial Research Organization, Australia), HadGEM2-ES (Met Office Dadley Centre, UK-Exeter), and MRI-CGCM3 (Meteorological Research Institute Coupled Ocean–Atmosphere General Circulation Model 3) in our analysis. The four GCMs were selected according to their higher resolution and established ability to replicate historical rainfall and temperature over the entire country compared to the other GCMs of CMIP5^26^. Changes in rainfall and temperature in mid- and end-century relative to the baseline were estimated based on GCM outputs. The adjusted formula for modified daily precipitation and temperature were expressed in Eqs. (1) and (2), respectively^41,57^.

1 $$P_{ adj,fur,d} = P_{ obs,d} \times \mathop \sum \limits_{i = 1}^{k} P_{i} \left( {\frac{{{\overline{\text{P}}}_{GCM.fur,m} }}{{\overline{P}_{GCM.ref,m} }}} \right),$$

where P_adj.fur,d_ is the adjusted daily rainfall for the future years, P_obs,d_ is the observed daily rainfall for the base years, ${\overline{\text{P}}}$. _GCM.fur,m_ is the monthly mean rainfall of the GCM outputs for the future years, ${\overline{\text{P}}}$. _GCM.ref.m_ is the monthly mean rainfall of the GCM outputs for the base years, P*i* is the weight of each grid cell, and *k* is the number of the grid cells.

2 $$T_{ adj,fur,d} = T_{ obs,d} \times \mathop \sum \limits_{i = 1}^{k} P_{i} \left( {{\overline{\text{T}}}_{GCM.fur,m} - \overline{T}_{GCM.ref,m} } \right),$$

where T_*adj.fur,d*_ is the adjusted daily temperature (maximum and minimum temperatures) for the future years, T_obs,d_ is the observed daily temperature for the base years, ${\overline{\text{T}}}$_*GCM.fur,m*_ is the monthly mean temperature of the GCM outputs for the future years, ${\overline{\text{T}}}$. _*GCM,ref.m*_ is the monthly mean temperature of the GCM outputs for the baseline, P*i* is the weight of each grid cell, and *k* is the number of the grid cells.

### Description of the crop simulation model

Decision Support Systems for Agrotechnology Transfer (DSSAT) version 4.7^58^ model was used to simulate maize grain yield using the baseline and future climate scenarios. In this study, the CERES-Maize model^39^ which is embedded within the DSSAT was used to simulate the phenology and yield of maize varieties, in response to climate and management factors. CERES-Maize is a process-based, management-oriented model that utilizes water, carbon, nitrogen (N) and energy balance principles to simulate the growth and development of maize. For each cultivar, the model runs with a daily time step and simulates crop growth, development and yield based on the effects of weather, soil characteristics and crop management practices^59^. A detailed description of the original CERES-Maize model can be found in Jones and Kiniry^60^ and Ritchie et al.^61^.

### Model inputs data

The DSSAT model requires field level input data including daily weather (minimum and maximum temperature, rainfall, and solar radiation), soil physical and chemical characteristics, crop variety parameters, and details of the crop management^58,62^. For this study, data on maize crop management (including planting date, plant density, fertilization and crop data) were obtained from the field trials conducted for the respective sites. Soil profile data of experimental stations were obtained from field measurements, the generic horizons of the profiles and soil types were classified using the FAO guidelines^63^. Daily rainfall, maximum and minimum temperature and solar radiation data of the experimental sites were obtained from an automated WatchDog weather station device (2000 Series, Spectrum Technologies, Aurora, IL, USA) located adjacent to the experimental sites. Maize varieties that are widely grown in all the study areas were used. The two maize varieties are SAMMAZ-16 (TZLComp1SynW–1) and SAMMAZ-26 (DTSTR WC1). These are improved and intermediate varieties developed by International Institute of Tropical Agriculture (IITA) in partnership with the Institute for Agricultural Research (IAR), Zaria, Nigeria, and have a combination of desired traits such as high grain yield and resistance to *Striga hermonthica*, in addition to these qualities, SAMMAZ-26 is considered as highly tolerant to drought^64^. The model calibration based on the crop management, soil physical, chemical, and hydrological properties had earlier been reported by Tofa et al.^53^.

### Model evaluation with an independent dataset

The comparison of simulated with observed yields allow the assessment of the model capacity to represent local cropping systems^41^. The independent data used for model evaluation included days to anthesis, days to physiological maturity, final grain yield and above ground biomass ha^−1^. These were collected from N response trials established at Iburu in SGS and Zaria in the NGS. Four field experiments were conducted during the rainy seasons of 2015 and 2016 each at Iburu and Zaria. Detailed climatic conditions of the two seasons were published in Tofa et al*.*^53^. The experiments were set up as a split plot design in randomized complete blocks with four N rates (0, 60, 120 and 180 kg ha^−1^) set as the main plot and the maize varieties were assigned to the sub-plots with three replications. The sub-plots contained four rows spaced 75 cm apart and 500 cm in length with intra-row spacing of 25 cm between stands which gave a plant density of 53,333 plants ha^−1^. Phosphorus (P) and potassium (K) were applied at the rate of 60 kg ha^−1^ each. Triple super phosphate (TSP) and muriate of potash (MOP) were used to supply the P and K fertilizers, respectively. Urea (46% N) was used as source for the four nitrogen (N) treatments. Half of the N and full rate of P and K were properly mixed and applied 10 days after sowing (DAS). The balance N was applied 45 DAS. The detailed climate, field observations, soil, and crop management practices used for model evaluation were published previously^53^. The response of the model was evaluated using three different statistical indicators, including root mean square error (RMSE), model estimation efficiency (EF) and index of agreement (d)^5,53^.

### Simulation protocol and analysis

The calibrated CERES-Maize model was used to access the impact of climate change on grain the yield of the two different maize varieties in the three agro-ecological zones of Nigeria. The effects of climate change on yield of each variety was simulated for 30-year baseline (1980–2009) weather data with the default atmospheric CO_2_ concentration of 380 ppm, while the future (2040–2069 and 2070–2099) climate scenarios were both simulated at 499 ppm CO_2_ (RCP4.5) and 571 ppm CO_2_ (RCP8.5). Other variables such as soil, cultivar and crop management practices are held constant. The seasonal analysis program in DSSAT was used to generate 30-year simulations and examine the variation in maize yield relative to the baseline. Two maize varieties, SAMMAZ-26 and SAMMAZ-16, were used which represents the drought-tolerant and non-drought-tolerant varieties, respectively.

The long-term simulations were done on an Alfisol soil from Kano representing the SS, a Typic Haplustalf from Zaria representing the NGS and a Ferric Luvisols from Abuja to represent the SGS. Nitrogen, soil water content, and organic matter content was allowed to be carried over between seasons, thereby not necessitating the need for re-initialization. The planting date was set at June 20 in the SS and June 30 for both NGS and SGS as recommended by Tofa et al.^53^. Plant population was set according to the national recommendation of 5.3 plants m^–2^. A constant inter row spacing of 75 cm and planting depth of 5 cm were maintained. For all simulated scenarios, the model was set up to supply the recommended rate of 120 kg N ha^−1^ using split application method. Half of N was set to apply at 10 days after sowing, and the other half at 45 days after sowing. Phosphorus and potassium were assumed to be non-limiting, so P and K sub-models were switched-off. A simple mathematical averaging was performed using excel to access the climate model ensemble mean. The impact of climate scenarios on maize yield was compiled and relative yield deviation from the baseline was computed according Faye et al.^65^ in Eq. (3):

3 $$\Delta {\text{Yield}} = \frac{{{\text{Yield}}_{scenario} - {\text{Yield}}_{baseline} }}{{{\text{Yield}}_{baseline} }} \times 100 \% ,$$

where ΔYield is the yield change due to climate change, Yield_*Scenario*_ and Yield_*baseline*_ are yields obtained under scenario and baseline weather conditions respectively.

Comparison was made for future water and N stress relative to baseline at each growing stage including emergence, end of juvenile, beginning of flowering, 75% silking, beginning of grain feeling, end of grain feeling and maturity. Values of the ensemble four GCMs were compared to the present for both varieties within each location to determine any difference in the actual water and N stresses in the model during the mid- and end-of-century using DSSAT output. The stress was measured on a scale of between 0 (minimum) and 1 (maximum) in the model each for water and N.

## Results and discussions

### Model calibration

Table 1 shows the values for genetic coefficients used in this study. The generated cultivar coefficients are within the range of DSSATv4.7 cultivar database. There was variation between the varieties in all the coefficients generated using Generalized Likelihood Uncertainty Estimation (GLUE). SAMMAZ-26 had higher potential kernel number (780 plant^–1^) than that of SAMMAZ-16 (743 plant^–1^). SAMMAZ-26 also recorded high thermal time from emergence to the end of juvenile phase (302 °C days) and from silking to physiological maturity (806 °C days) and high optimum kernel filling rate (6.5 mg day^–1^) and thermal time between successive leaf tip appearances (40 °C days). However, the delay in development for each hour that day-length is above 12.5 h was slightly higher (0.424 days) for SAMMAZ-16. The parameterized CERES‐Maize model was used to assess the accuracy of cultivar coefficients by simulating days to anthesis, days to physiological maturity, grain yield kg ha^–1^, and above ground biomass kg ha^–1^ for the two maize varieties (Table 2). There was high prediction accuracy for all the tested parameters as indicated by low RMSE and high d-index values for the two varieties. Excellent statistics were achieved for calibration of the phenological parameters (anthesis and maturity) with RMSE below 3 days and d-index above 0.92 for of both varieties. For SAMMAZ-16, the RMSE between the observed and simulated values of grain yield and above ground biomass, were 245 kg ha^–1^ and 1152 kg ha^–1^, respectively, with corresponding d-index of 0.91 and 0.80. For variety SAMMAZ-26, the RMSE for grain yield was 157.7 kg ha^–1^ with d-index 0.61 while for above ground biomass the RMSE was 784 kg ha^–1^ with d-index 0.88. This result suggests accurate predictions of all parameters for model application.

**Table 1:** Genetic coefficients for SAMMAZ-16 and SAMMAZ-26 maize varieties used in the study (coefficients for SAMMAZ-16 were adapted from Tofa et al.^55^).

Code	Description	SAMMAZ-16	SAMMAZ-26
P1	Thermal time from emergence to the end of juvenile phase (degree days)	253.3	302.0
P2	Delay in development for each hour that day-length is above 12.5 h (days)	0.424	0.400
P5	Thermal time from silking to physiological maturity (degree days)	794.9	805.9
G2	Maximum possible number of kernels per plant	743.3	780.0
G3	Kernel optimum filling rate during the linear grain filling stage optimum conditions (mg/day)	6.25	6.50
PHINT	(Phylochron interval): thermal time between successive leaf tip appearances (degree days)	38.90	40.00

**Table 2:** Simulated and observed mean values for anthesis, physiological maturity, grain yield, and above ground biomass with their respective statistical indices for the calibrated maize varieties (values for SAMMAZ-16 Adapted from Tofa et al.^55^).

Parameter	Number of experiments	Simulated	Observed	RMSE	d-Index
**SAMMAZ-15**					
Number of days to anthesis	14	55.4	56.4	1.9	0.93
Number of days to maturity	14	100.2	100.4	2.0	0.97
Grain yield at harvest (kg ha^–1^)	14	5253	5272	245	0.91
Above ground biomass (kg ha^–1^)	14	15,606	14,990	1152	0.80
**SAMMAZ-26**					
Number of days to anthesis	9	60	60	2.1	0.93
Number of days to maturity	9	105	106	2.0	0.97
Grain yield at harvest (kg ha^–1^)	9	5231	5223	157.7	0.61
Above ground biomass (kg ha^–1^)	9	16,123	15,525	784.4	0.88

### Model evaluation

The model evaluation results using an independent dataset showed that the model accurately predicted days to anthesis and maturity with low RMSE values of < 2 days for both parameters with high d-index and prediction deviations (PDs) below 2% for both varieties (Table 3). Similarly, grain yield was adequately simulated with low RMSE of 730 kg ha^−1^ for SAMMAZ-16 and 725 kg ha^−1^ for SAMMAZ-26 with PDs below 6%. The comparison of predicted and observed above ground biomass for both varieties indicted good performance. However, the variety SAMMAZ-26 had the least statistical errors with RMSE 1990 kg ha^−1^ and d-index value of 0.95. Higher model efficiency (EF) values were estimated with variety SAMMAZ-26 for all parameters compared to SAMMAZ-16 variety (Table 3). We found the model’s performance satisfactory based on the closeness of fit between observed and simulated parameters evaluated, thereby suggesting that the model is robust and near accurate to make wider assessments across different environments under study. These results are in agreement with previous findings from the same agro-ecologies of Nigeria^5,53,66,67^.

**Table 3:** Comparisons of simulated and observed mean values on days to anthesis, days to maturity, grain yield, and above ground biomass for model evaluation with their respective statistical indices for the calibrated maize varieties.

Parameter	Number of experiments	Simulated	Observed	Prediction deviation ^(^%)	RMSE	d-index	EF
**SAMMAZ-16**							
Number of days to anthesis	4	59	58	1.7	1.03	0.67	− 0.10
Number of days to maturity	4	104	104	0.0	1.39	0.90	0.56
Grain yield at harvest (kg ha^–1^)	4	3414	3278	4.1	750	0.95	0.79
Above ground biomass (kg ha^–1^)	4	10,471	8977	16.6	2535	0.91	0.45
**SAMMAZ-26**							
Number of days to anthesis	4	62	62	0.0	1.94	0.83	0.30
Number of days to maturity	4	108	109	− 0.9	1.28	0.97	0.89
Grain yield at harvest (kg ha^–1^)	4	3538	3752	− 5.7	725	0.96	0.83
Above ground biomass (kg ha^–1^)	4	10,244	10,058	1.8	1990	0.95	0.70

### Projected trends in minimum and maximum temperatures

**Figure 2:**
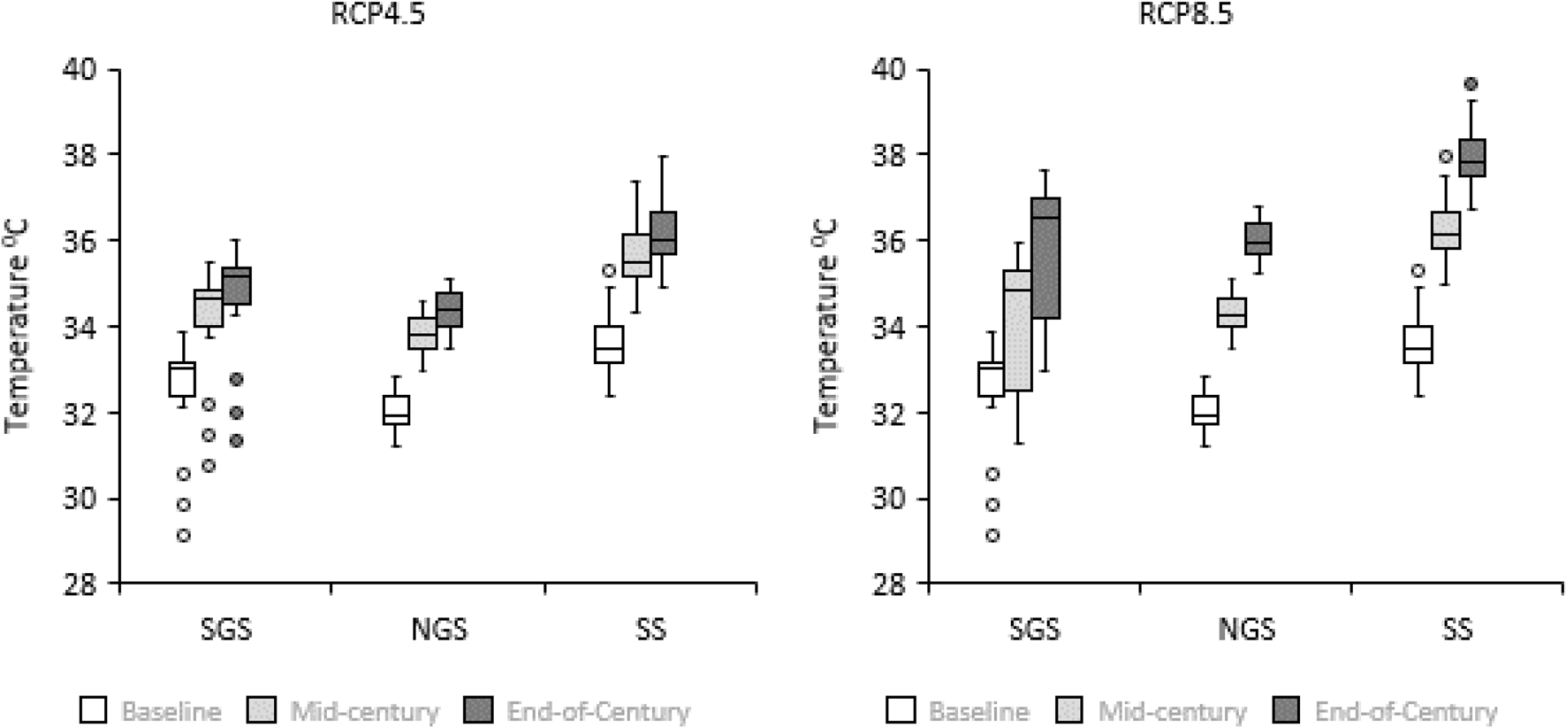
Thirty years seasonal maximum temperature for baseline (1980–2009) and ensemble mid–century (2040–2069) and end of century (2070–2099) periods under RCP4.5 (left) and RCP8.5 (right) in SGS, NGS and SS. Extremes on whiskers (two lines outside the box) encompass the range between lowest and highest values, the box spans the interquartile range, extremes of the box encompass the range between the 25 and 75% quartiles, and the horizontal line within each box section shows the median. Circles located outside the whiskers referred to as outliers.

**Figure 3:**
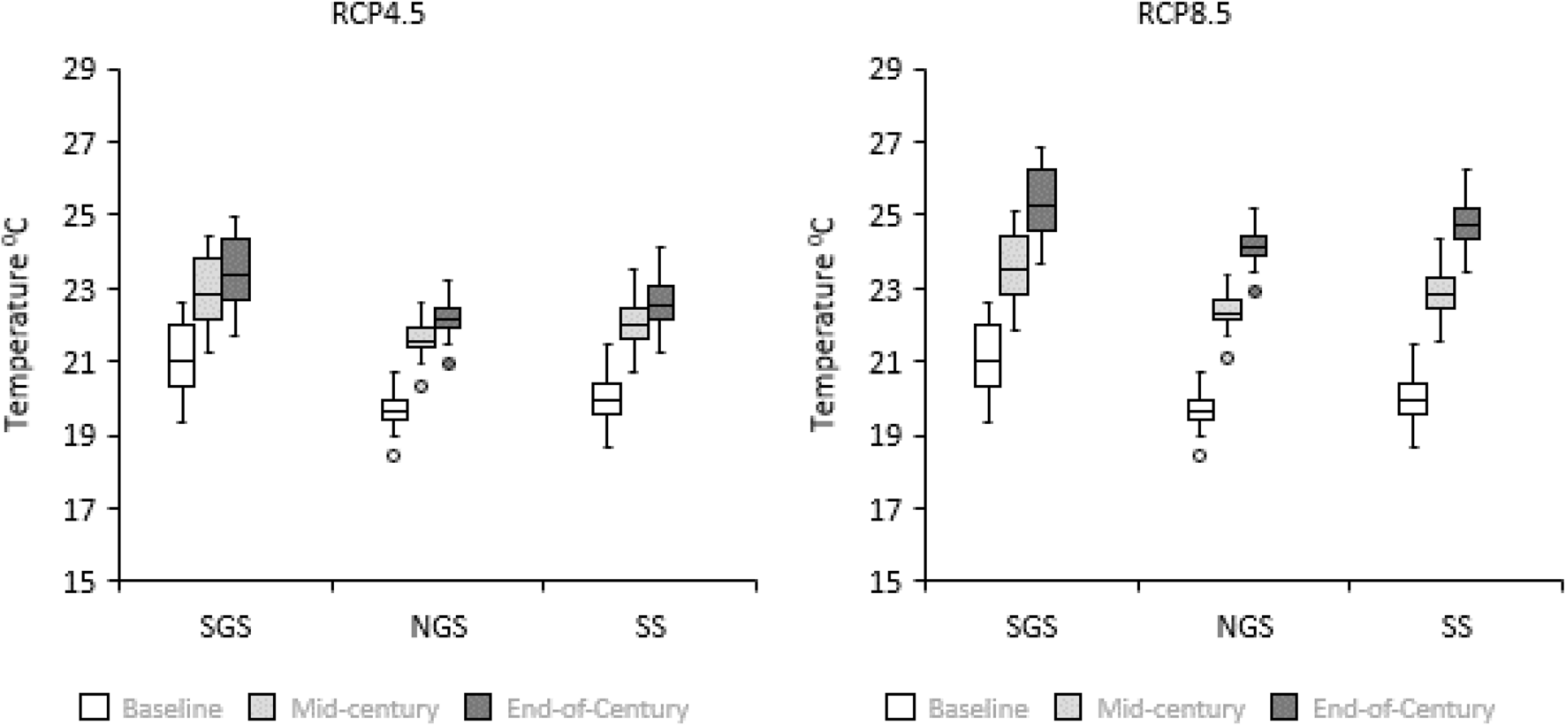
Thirty years seasonal minimum temperature for baseline (1980–2009) and ensemble mid–century (2040–2069) and end of century (2070–2099) periods under RCP4.5 (left) and RCP8.5 (right) in SGS, NGS and SS. Extremes on whiskers (two lines outside the box) encompass the range between lowest and highest values, the box spans the interquartile range, extremes of the box encompass the range between the 25 and 75% quartiles, and the horizontal line within each box section shows the median. Circles located outside the whiskers referred to as outliers.

**Figure 4:**
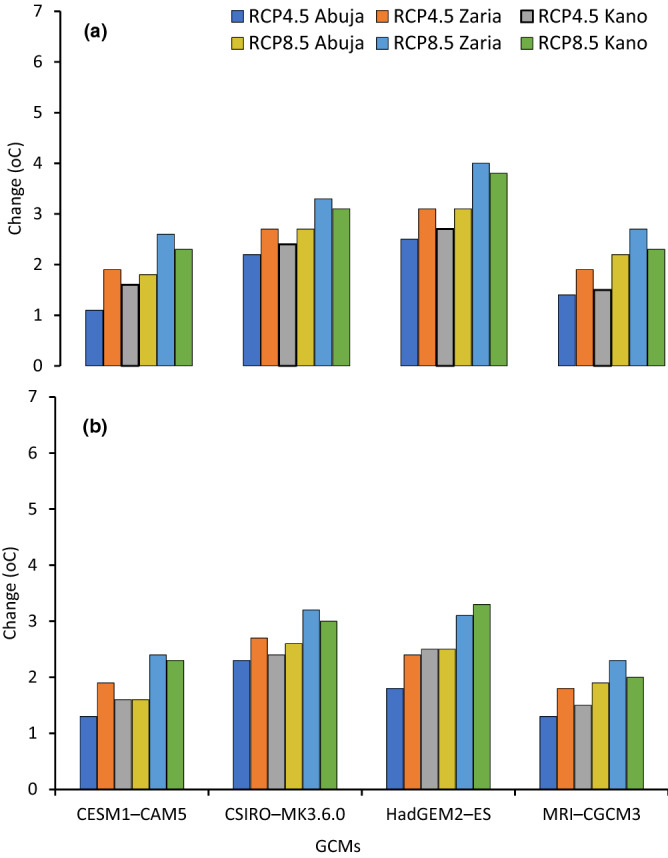
Minimum (**a**) and maximum (**b**) temperature change from the baseline period for four GCMs under RCP4.5 and RCP8.5 at Abuja, Zaria and Kano in Nigeria for mid-century period.

**Figure 5:**
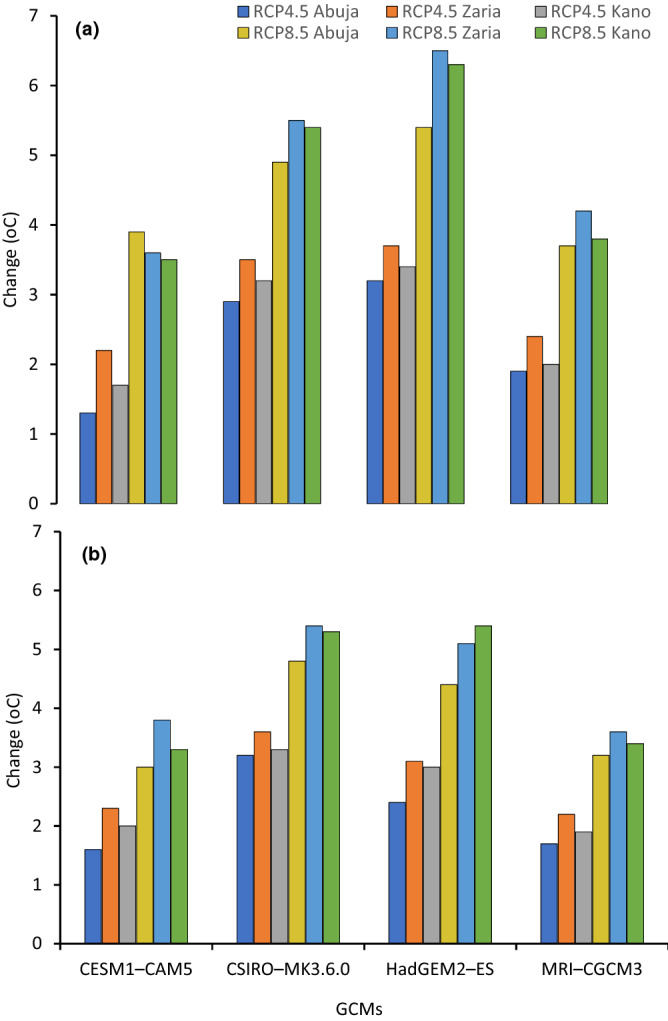
Minimum (**a**) and maximum (**b**) temperature change from the baseline period for four GCMs under RCP4.5 and RCP8.5 at Abuja, Zaria and Kano in Nigeria for end-of-century period.

The thirty-year temperature projection under the medium (RCP 4.5) and a high (RCP 8.5) emission scenarios for the future climates (mid- and end-century) in the three locations indicated significant increases in temperatures, compared to baseline (Figs. 2, 3). All GCMs show increases as indicated by the maximum and minimum temperatures for all the four GCMs (Figs. 4, 5). Multi-model GCM ensemble projections have been recommended for climate change impact studies to take care of uncertainties embedded within GCMs^68^. Based on the GCM ensemble outputs (Table 4), both the minimum and maximum temperatures increase gradually in time from the mid-century period to the end of the century in all locations. However, the RCP8.5 scenario projected the highest increase in temperature compared to RCP4.5 within each period for all locations.

**Table 4:** Baseline and simulated ensemble annual climatic changes for rainfall, minimum and maximum temperatures for mid-century and end-of-century under RCP4.5 and RCP8.5 relative to the baseline data in SGS, NGS and SS of Nigeria.

	Abuja in SGS				Zaria in NGS				Kano in SS			
	Rainfall		*t*_*min*_ °C	*t*_*max*_ °C	Rainfall		*t*_*min*_ °C	*t*_*max*_ °C	Rainfall		*t*_*min*_ °C	*t*_*max*_ °C
	(mm)	%			(mm)	%			(mm)	%		
**Baseline**												
	1540.8	–	21.1	32.4	998.1	–	19.2	31.6	752.8	–	20.0	33.7
**Mid-century**												
RCP4.5	18.68	1.23	1.80	1.68	18.38	1.85	2.40	2.20	52.33	6.95	2.05	2.00
RCP8.5	0.15	0.03	2.45	2.15	29.78	3.00	3.15	2.75	80.15	10.63	2.88	2.65
**End-of-century**												
RCP4.5	31.10	2.00	2.33	2.23	21.28	2.13	2.95	2.80	49.95	6.65	2.58	2.55
RCP8.5	50.13	3.28	4.48	3.85	66.08	6.63	4.95	4.48	151.38	20.10	4.75	4.35

In Abuja in the SGS, the minimum temperature is projected to increase by 1.8 °C in the mid-century and 2.3 °C by the end-century under RCP4.5, meanwhile under RCP8.5 minimum temperature will rise by 2.5 °C in the mid-century and 4.5 °C by the end-century compared to baseline. The maximum temperature was projected to increase by 1.7 and 2.23 °C in the mid- and end-century, respectively, compared to the baseline period under the RCP4.5 scenario. Under the RCP8.5 scenario, the maximum temperature was expected to increase by 2.2 and 3.9 °C in the mid- and end-century periods, respectively.

In Zaria in the NGS, the minimum and maximum temperatures will increase by 2.4 and 2.2 °C in the mid-century, and 3.0 and 2.8 °C at the end-century under RCP4.5 compared to baseline period. Under high emission scenario (RCP8.5), the minimum and maximum temperatures were projected to increase by 3.2 and 2.8 °C in the mid-century, and increase by 5.0 and 4.5 °C by the end of century compared to baseline period.

In Kano in the SS, the projections under RCP 4.5 indicates minimum and maximum temperatures will increase by 2.1 and 2.0 °C in the mid-century and by 2.9 and 2.6 °C by end-century, respectively. Under RCP 8.5, the minimum and maximum temperatures will increase by 2.9 and 2.7 °C, respectively in the mid-century while projected increase by end-century will be 4.8 and 4.4 °C for minimum and maximum temperatures, respectively. These results agree with previous reports that indicated future warming of the air in the different parts of Nigeria^20,21,25,31^. The temperature increases in our study fall within the ranges reported by Niang et al.^69^, who projected temperature increase to exceed 2 °C by 2050 across much of Africa and to reach between 3 and 6 °C by the end of the century. Our results showed that the temperature changes vary spatially and increase from the SGS to the SS. This temperature distribution is consistent with those predicted for Nigeria by Abiodun et al.^70^. They reported increase in temperature in all the ecological zones, with the higher value between 1 and 4 °C in the SS.

In all the study areas, the minimum temperature increases faster than that of the maximum temperature under both RCPs. Our findings agreed with Dike et al.^71^ who found that the magnitude of the increasing trend of minimum temperature is higher when compared with the increasing trend in maximum temperature over Nigeria. They stated that, minimum temperature increased at a rate of 0.51 °C per decade during 1971–2013, which by far exceeds the 0.17 °C per decade increase in maximum temperature. Analysis by Meehl et al.^72^ revealed that daily minimum temperatures will increase more rapidly than daily maximum temperatures leading to the increase in the daily mean temperatures and a greater likelihood of extreme events and these changes could have detrimental effects on grain yield. High night temperature (minimum temperature in the range of 22–25 °C) could contribute to the lower yield of maize crops grown in the humid tropics because of higher rate of respiration^73^. Nightly weight losses by whole plant averaged 40% of daily weight gain^73^. According to Hatfield^74^, maize grain yields decreased from 84 to 100% because of exposure to high night time temperatures and disruption of the pollination process as evidenced by the large reduction in kernels per ear. During the grain-filling period, however, exposure to higher night temperatures shortened the grain-filling period by increasing the rate of senescence^74^.

### Projected changes in seasonal rainfall

**Figure 6:**
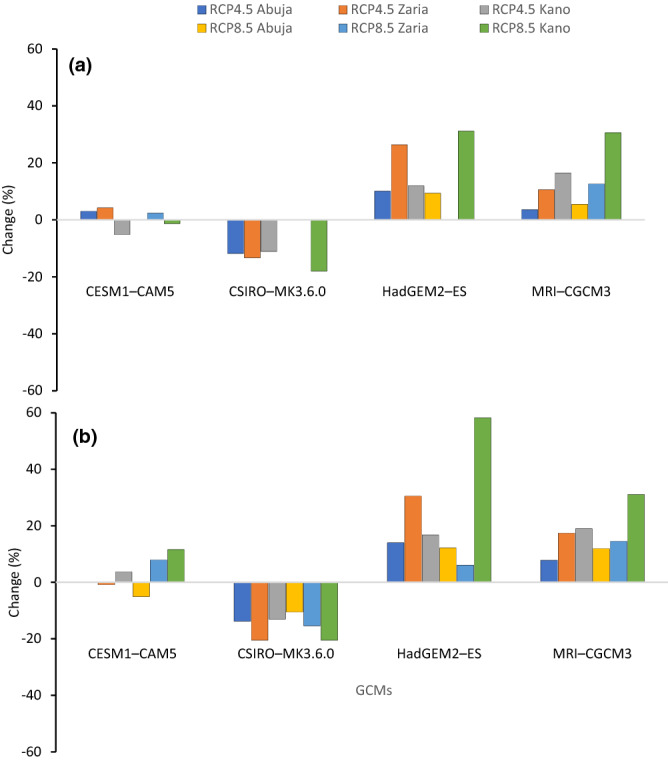
Rainfall change in percentage from the baseline period for four general circulation models under RCP4.5 and RCP8.5 at Abuja, Zaria and Kano in Nigeria for mid-century (**a**) and end-of-century (**b**) periods.

Figure 6 presents the seasonal changes in rainfall computed from baseline to future climates for each GCM under both RCP4.5 and 8.5 scenarios. Based on the ensemble GCMs (Table 4), the seasonal rainfall is expected to increase in all study areas, for both RCPs, with the higher increases in the SS. The change in rainfall, increases in time from mid- to end-of-century in all the locations under RCP8.5. However, under the RCP4.5 scenario, the rainfall increase is limited with the slight decrease (0.3%) for SS from mid-century to the end of century. With the ensemble GCM, the largest projected mean rainfall increases of 10.6% in the mid-century and 20.1% in the end of century were expected in the SS zone under RCP8.5. Under both RCP4.5 and RCP8.5 an increase in rainfall is consistently projected to be lower for SGS during both the mid- and end-century periods. In this agro-ecology, the increase in rainfall ranged between 0.03% in the mid-century and 3.28% in the end-century, both under RCP8.5. The study by Shiru et al.^75^ also reported a significant increase in seasonal rainfall in the range of 0–20% in most parts of the northern Nigeria. Adhikari et al.^76^ observed that large uncertainty exists in projecting precipitation, and changes would range from − 15 to + 27% by 2060s in Africa. This projected increase in rainfall in the Guinea and Sudan savanna may pose a risk for annual flooding which may affect crop performance.

### Projected impact of climate change on maize yield

Tables 5 and 6 shows the simulated maize yield with the baseline (1980–2009) and relative yield change in the mid-century (2040–2069) and end-century (2070–2099) under RCP4.5 and RCP8.5 scenarios. Irrespective of variety, the simulated ensemble of four GCMs showed a consistent decline in maize yield for both future climates relative to the baseline period in all locations. The two maize varieties performed differently in each agro-ecological zone. In SGS, relative to the baseline, the grain yield of SAMMAZ-26 is expected to decrease by 14 and 16% under RCP4.5 and RCP8.5, respectively, in the mid-century. The declines are 17% (RCP4.5) and 37% (RCP8.5) by the end of century. The grain yield of SAMMAZ-16 will decrease by 15% under RCP4.5 and 19% under RCP8.5 in the mid-century. Yield decrease of 21% (RCP4.5) and 42% (RCP8.5) are projected for the end of century. Future maize grain yields simulated for the NGS also shows consistent differences between the two varieties. Grain yield of SAMMAZ-26 will decrease by 9% under RCP4.5 and 14% under RCP8.5 during the mid-century. The decrease will be 13% under RCP4.5 and 32% under RCP8.5 by the end of the century period. For variety SAMMAZ-16, the yields are expected to decrease by 13 and 19%, under RCP4.5 and RCP8.5, respectively, in the mid-century. By the end of century yield will decrease by 18% under RCP4.5 and 38% under RCP8.5. In SS, the results shows that future yields of maize also differ strongly between the two varieties. Relative to the baseline climate, grain yield of SAMMAZ-26 will reduce by 18% under RCP4.5 and 25% under RCP8.5 in the mid-century. For end-of-century yield will reduce by 23% and 43% under RCP4.5 and RCP8.5, respectively. For SAMMAZ16, the yield is expected to decrease by 19% for RCP4.5 and 28% for RCP8.5 in mid-century. The estimated yield reduction of SAMMAZ-16 is 26% under RCP4.5 and more than 46% for RCP8.5 by the end of century.

**Table 5:** Baseline and simulated grain yield (kg ha^–1^) of different maize varieties and the relative yield change (%) at mid-century in three agro-ecologies of Nigeria.

		Baseline (1980–2009)	RCP4.5		RCP8.5	
			GCMs ensemble	Relative yield change	GCMs ensemble	Relative yield change
Location	Variety	kg ha^–1^		(%)	kg ha^–1^	(%)
Abuja in SGS	SAMMAZ-16	4242	3592	− 15	3406	− 20
	SAMMAZ-26	4593	4050	− 14	3837	− 16
Zaria in NGS	SAMMAZ-16	4791	4159	− 13	3890	− 19
	SAMMAZ-26	4859	4417	− 9	4172	− 14
Kano in SS	SAMMAZ-16	4606	3724	− 19	3323	− 28
	SAMMAZ-26	4383	3612	− 18	3287	− 25

**Table 6:** Baseline and simulated grain yield (kg ha^–1^) of different maize varieties and the relative yield change (%) at end-century in three agro-ecologies of Nigeria.

		Baseline (1980–2009)	RCP4.5		RCP8.5	
			GCMs ensemble	Relative yield change	GCMs ensemble	Relative yield change
Location	Variety	kg ha^–1^		(%)	kg ha^–1^	(%)
Abuja in SGS	SAMMAZ-16	4242	3351	− 21	2479	− 42
	SAMMAZ-26	4593	3806	− 17	2896	− 37
Zaria in NGS	SAMMAZ-16	4791	3955	− 18	2995	− 38
	SAMMAZ-26	4859	4245	− 13	3291	− 32
Kano in SS	SAMMAZ-16	4606	3434	− 26	2444	− 47
	SAMMAZ-26	4383	3392	− 23	2519	− 42

Higher percentage reduction in maize yield was predicted by the end of century compared to the middle future climate. Ensemble grain yield decline was between 9 and 19% under RCP4.5 and between 14 and 28% under RCP8.5 in the mid–century. In the end of century, yield reductions were between 13 and 26% under RCP4.5 and 32–47% under RCP8.5. Thus, ensemble grain yield change of between − 9 and − 47% in our study falls within the wide ranges (− 98 to + 16%) of climate change impact reported for SSA^77^. Roudier et al.^78^ found that the response of crop yield to climate change in West Africa can vary from − 50 to + 90% in a selection of 16 publications. This range is even wider in the review made by Müller et al.^79^ which showed that projected impacts relative to current African production levels range from − 100 to + 168%.

**Figure 7:**
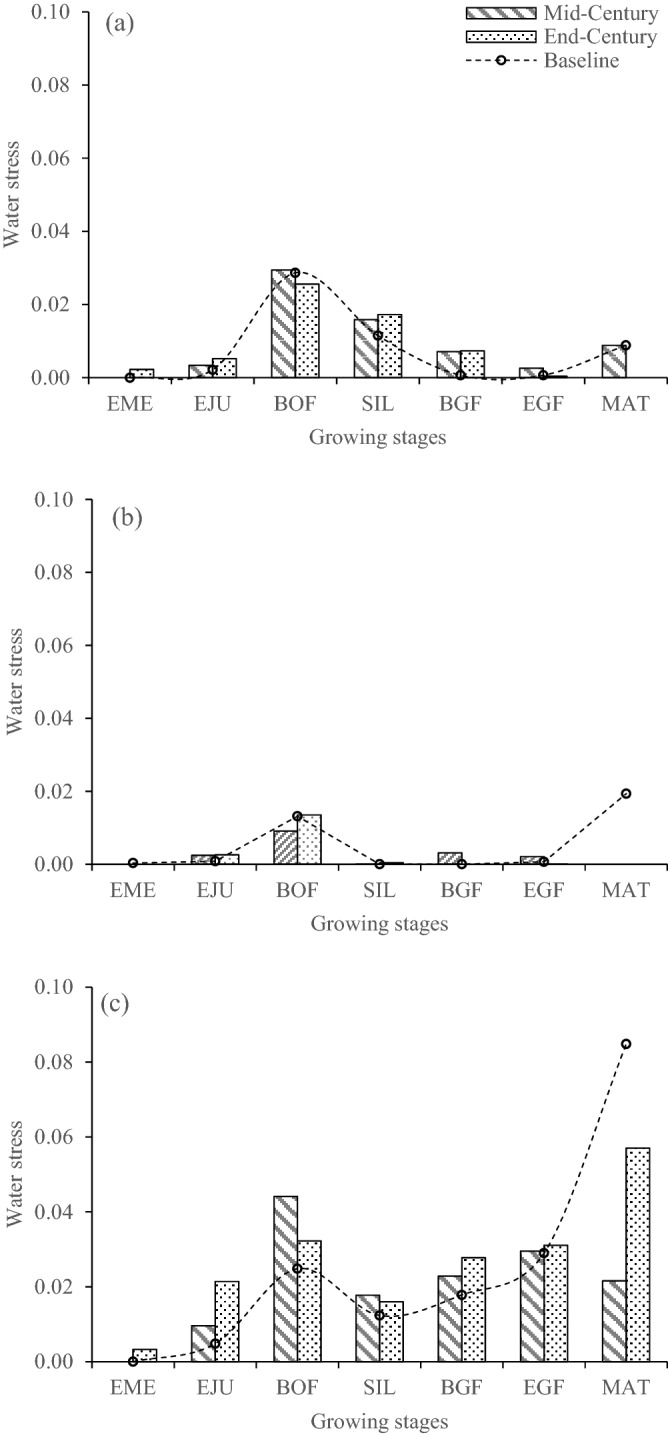
Comparison of baseline and future model water stress at each growing stage: Abuja in the SGS (**a**), Zaria in the NGS (**b**) and Kano in the SS (**c**) using DSSAT output. (For mid-and -end-centuries, the stresses were from four GCMs within each location). *EME* emergence, *EJU* end of juvenile, *BOF* beginning of flowering, *SIL* 75% silking, *BGF* beginning of grain feeling, *EGF* end of grain feeling and *MAT* maturity.

**Figure 8:**
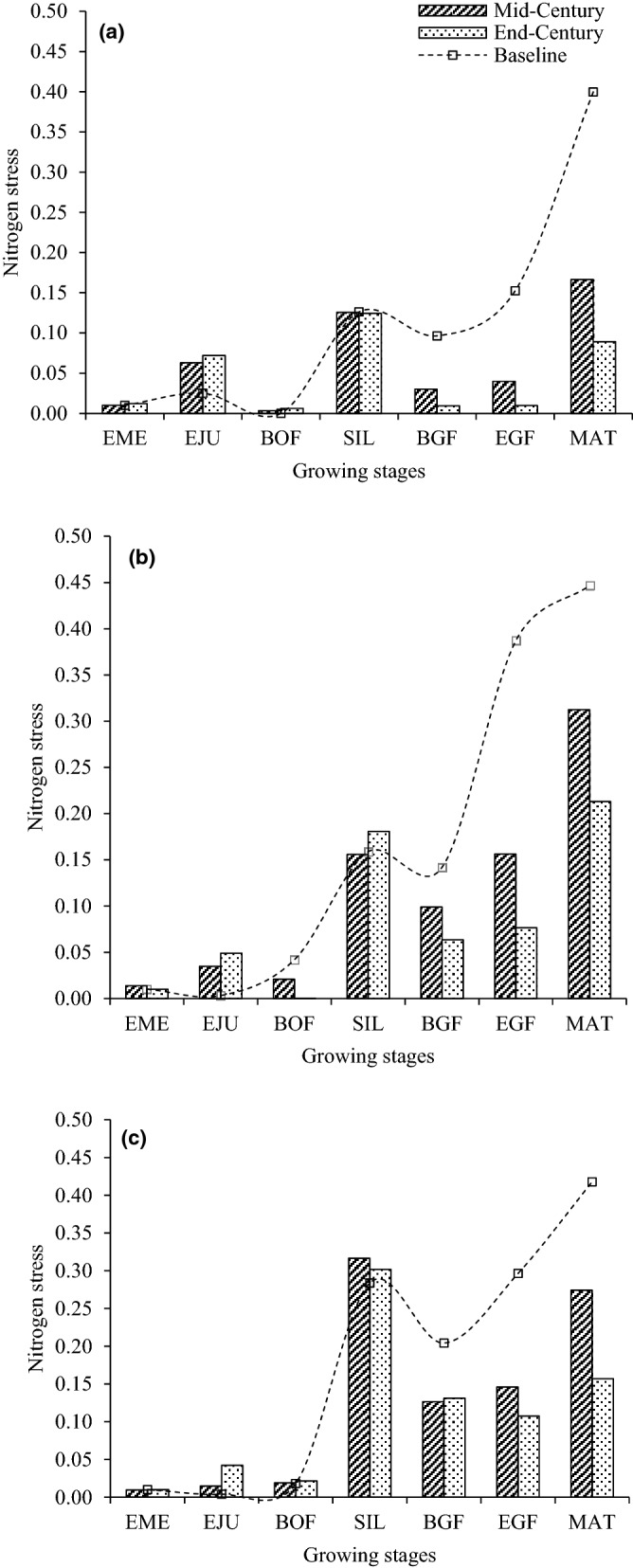
Comparison of baseline and future model nitrogen stress at each growing stage: Abuja in the SGS (**a**), Zaria in the NGS (**b**) and Kano in the SS (**c**) using DSSAT output. (For mid-and -end-centuries, the stresses were from four GCMs within each location). *EME* emergence, *EJU* end of juvenile, *BOF* beginning of flowering, *SIL* 75% silking, *BGF* beginning of grain feeling, *EGF* end of grain feeling and *MAT* maturity.

Based on the comparative study of water and N stress under current and future climate change scenarios (Figs. 7, 8), the effects of future water and N stress on the crop were determined to be less throughout the growing season. This could be due to the increase in rainfall predicted in this study (Tables 5, 6) which is anticipated to significantly reduce water stress and enhance N uptake on maize production in both future climates. The larger reduction in grain yield in the late future production system could be from the intensified heat stress due to increases in maximum temperature (ranged 3.85–4.48 °C) and minimum temperature (ranged 4.48–4.95 °C) across the locations. This agrees with previous reports that indicated the greatest reduction in maize yield is due to increase in temperature than a decrease in rainfall^33,40,68,80–82^. A study by Tesfaye et al.^33^ under hotter and drier climate change scenarios in South Africa showed significant yield reductions of local maize varieties by 21, 33 and 50% when temperature increased by 1, 2 and 4 °C, respectively. Similarly, Lobell and Burke^80^ showed that an increase in temperature by 2 °C would result in a greater reduction in maize yields than a decrease in precipitation by 20%. Traore et al.^40^ also estimated maize grain yield reductions of between 51 and 57% under current farmers’ practices for southern Mali. In Nigeria, Abiodun et al.^70^, stated that the warming increases with latitudes, with the lowest warming over the southern region and the highest at the northern region. The southern regions receive lower warming than the interior because the cooling effect from the Atlantic Ocean reduces the warming near the coast. Hence, the northern stations are expected to be warmer than southern stations, therefore, more yield reduction in the Sudan savanna.

Grain yield reduction due to climate change vary spatially over the three study areas. Under both RCPs, the effect was more pronounced in the SS in both mid- and end-centuries, while the least decline in yield was observed in the NGS where the predicted increase in temperature was higher under both RCPs in two centuries. This is probably due to the better soil condition in the NGS that could reduce the adverse effect of the higher temperatures. Similarly, Sowunmi and Akintola^83^, concluded that Guinea savannah zone is suitable for maize production regarding good soil and temperature. In contrary, another study in Nigeria indicated that yield reduction increases from the southern Guinea savanna to the Sudan savanna. They projected that an increase in temperature by 4 °C reduced maize yields by 11.7%, 19.7% and 21.6% in the SGS, NGS and Semi-Arid, respectively, at 330 ppm CO_2_ concentration^2^.

Under both RCPs, the results shows that, the predicted temperature increase is likely to have the biggest adverse effect on yields of both varieties at the end of the century in all the three study sites. However, the simulated reduction in grain yield would be lower using SAMMAZ-26 in both climate change scenarios compared to the SAMMAZ-16. This suggests that the drought-tolerant variety would perform better at both future climate periods compared to non-drought-tolerant variety. Our results are consistent with findings of Cairns et al.^36^ who reported that drought tolerant varieties are more resilient to the effects of high temperature due to climate change in SSA.

The high yield reductions in the range of − 13 to − 23% under RCP4.5 and − 32 to − 42% under RCP8.5 observed for the drought-tolerant variety by the end of the century across the study areas highlighted the need to breed maize varieties and hybrids that combine tolerance to drought and heat stress. This is because drought and heat stress occur simultaneously in farmers’ fields. The breeding strategies in maize that had been adopted so far have focused on improvement of each stress separately^28^. Cairns et al.^36^ suggested that the genetic control of drought, heat, and combined drought and heat tolerance are largely independent of each other. Preliminary results of maize evaluated under the Drought Tolerant Maize for Africa (DTMA) project revealed that tolerance to drought stress does not necessarily confer tolerance to heat stress or combine drought and heat stress, which have implications to breed for adaptation to climate change in maize production systems in SSA^36^. This is because higher temperatures are often associated with increases in evapotranspiration and a reduction in soil moisture levels which hasten the onset and severity of drought stress, especially in rainfed drylands^33,84^. This study suggests that breeding maize for combine drought and heat stress could be an effective adaptation strategy in dealing with the adverse effects of high temperature in the future climate change.

## Conclusion

This study evaluated the impacts of climate change on maize production in northern Nigeria in two future time periods (mid- and end-centuries) compared to a baseline period (1980–2009), under RCPs 4.5 and 8.5 climate change emission scenarios. The climate projections for major maize growing zones in Nigeria showed rise in temperatures. Our climate modelling results suggest that with ensemble GCM, both the minimum and maximum temperatures will increase from the mid-century period to the end of the century in all locations. The annual mean temperature under RCP8.5 scenario was the highest compared with RCP4.5 within each century period for all locations. Rainfall is expected to increase at all the study areas, for both RCPs, with the higher increases of 10.6% in the mid-century and 20.1% in the end of century in the SS. As projected by the ensemble GCMs in combination with 2 RCPs and DSSAT model, climate change will result in reduction of yield of between 9 and 28% for the mid-century (2040–2069) and between 13 and 47% for end of the century (2070–2099), depending on the environment and variety, with farmers in the Sudan savanna environment having more negative impact. Results shows that the use of improved and drought-tolerant variety as adaptation strategy can reduce the negative impact of climate change. On average, the yield difference between SAMMAZ-26 and SAMMAZ-16 is 2% under RCP4.5 and up to 4% under RCP8.5 during the mid–century, while in the end of century, the average yield difference between the two varieties is 4% and 5% under RCP4.5 and RCP8.5, respectively, across the three study areas. Though the yield reduction due to climate change was less for the drought-tolerant variety compared to the non-drought tolerant variety, the high yield reduction of the drought-tolerant variety suggests that breeders should develop varieties that combine tolerance to drought with that of heat to withstand the impacts of increased temperatures in the agro-ecological zones of northern Nigeria.

## Data Availability

The datasets generated during and/or analysed during the current study are available from the corresponding author on reasonable request.
